# Reclassification of 11 Members of the Family Rhodobacteraceae at Genus and Species Levels and Proposal of *Pseudogemmobacter hezensis* sp. nov.

**DOI:** 10.3389/fmicb.2022.849695

**Published:** 2022-04-13

**Authors:** Tengfei Ma, Han Xue, Chungen Piao, Chengyi Liu, Mei Yang, Danran Bian, Yong Li

**Affiliations:** ^1^Key Laboratory of Forest Protection of National Forestry and Grassland Administration, Ecology and Nature Conservation Institute, Chinese Academy of Forestry, Beijing, China; ^2^Panzhihua City Academy of Agricultural and Forest Sciences, Panzhihua, China

**Keywords:** *Xinfangfangia*, *cereibacter*, *Rhodobacter*, *Tabrizicola*, *Pseudotabrizicola*, GTDB-Tk, average amino acid identity

## Abstract

A novel Gram-stain-negative, aerobic, motile bacterial strain, D13-10-4-6^T^, was isolated from the bark sample of *Populus* × *euramericana*. The strain could grow at 15–35°C, at pH 6–10 and in 0–4% (w/v) NaCl, and the strain tested positive for oxidase and catalase activities. The main polar lipids were phosphatidylmonomethylethanolamine, diphosphatidylglycerol, phosphatidylglycerol, and phosphatidylethanolamine. The main respiratory quinone was Q-10, and the predominant fatty acid was C_18:1_ ω7*c*. The phylogenetic analyses showed that the strain belonged to the genus *Pseudogemmobacter* of the family Rhodobacteraceae. The family Rhodobacteraceae is an ecologically diverse group that includes bacteria from aquatic to terrestrial ecosystems. As a consequence, the classification of the family Rhodobacteraceae is difficult, not least when the early taxonomy work relied heavily on 16S rRNA gene analysis. Recently, the taxonomic status of many members of the family has been revised based on the genome analysis; however, there are still some classification conflicts due to the lack of genome sequences and parallel publication time. In this study, phylogenetic trees based on 16S rRNA gene, *gyr*B gene, and 120 concatenated proteins, the average amino acid identity (AAI) and percentage of conserved proteins (POCP) have been used for the analysis of strain D13-10-4-6^T^ and other members of 15 genera within the family to further clarify their taxonomic relationships. For the data of phylogeny, AAI, and POCP, the taxonomic proposals are (1) reclassification of *Rhodobacter tardus* as the type species of a novel genus, *Stagnihabitans* gen. nov., as *Stagnihabitans tardus* comb. nov.; (2) reclassification of *Tabrizicola alkalilacus*, *Tabrizicola sediminis*, *Tabrizicola algicola* into a novel genus, *Pseudotabrizicola* gen. nov., as *Pseudotabrizicola alkalilacus* comb. nov., *Pseudotabrizicola sediminis* comb. nov., *Pseudotabrizicola algicola* comb. nov.; (3) reclassification of *Rhodobacter sediminicola* into the genus *Cereibacter* as *Cereibacter sediminicola* comb. nov.; (4) reclassification of *Rhodobacter flagellatus*, *Rhodobacter thermarum*, and *Xinfangfangia soli* into the genus *Tabrizicola* as *Tabrizicola flagellatus* comb. nov., *Tabrizicola thermarum* comb. Nov., and *Tabrizicola soli* comb. nov.; (5) reclassification of *Xinfangfangia humi* into the genus *Pseudogemmobacter* as *Pseudogemmobacter humicola* comb. nov.; (6) classification of strain D13-10-4-6^T^ as a novel species of the genus *Pseudogemmobacter*, for which the name *P. hezensis* sp. nov. is proposed, the type strain is D13-10-4-6^T^ (= CFCC 12033^T^ = KCTC 82215^T^).

## Introduction

*Populus* × *euramericana* cakers on poplar trees were found in China for many years, and the stem or branch bark of the diseased tree was cracked and exuded frothy fluid. During our investigation of the bacterial diversity in *Populus* × *euramericana* caker, strain D13-10-4-6^T^ was isolated from the symptomatic bark of *Populus* × *euramericana* caker. The phylogenetic analyses showed that the strain belonged to the genus *Pseudogemmobacter* of the family Rhodobacteraceae. The family Rhodobacteraceae, described by Garrity et al. (2005), the so-called purple non-sulfur bacteria (Imhoff et al., 1998), is one of the major subdivisions of the class Alphaproteobacteria. It is ecologically and phenotypically diverse, and most of the members of the family have been found in various marine environments, including seawater, sea sediments, sea ice, coastal biofilms, marine animal tissues, and seaweeds (Selje et al., 2004; Buchan et al., 2005; Brinkhoff et al., 2008). At the time of writing, the family included more than 180 genera with validated names.^1^

The early classification of the genera within the family Rhodobacteraceae relied heavily on the analysis of 16S rRNA gene sequence and resulted in several non-monophyletic genera, for instance, the genus *Rhodobacter* (Imhoff et al., 1984). The genus *Rhodobacter* was reclassified by Suresh et al. (2019) and Hördt et al. (2020) based on the genome analysis. The members of the genus were divided into five distinct clades in the 16S rRNA gene-based phylogenetic tree constructed by Suresh et al. (2019). The *Cereibacter sphaeroides* (formerly *Rhodobacter sphaeroides*) clade was reclassified into the genus *Luteovulum* (Suresh et al., 2019) and then transferred into the genus *Cereibacter* by Hördt et al. (2020). However, *Rhodobacter alkalitolerans*, which belongs to the *C. sphaeroides* clade, was not reclassified due to the lack of genomic sequence. At present, the genus *Rhodobacter* contains 13 species with validated names according to the List of Prokaryotic names with Standing in Nomenclature (LSPN).^2^ The taxonomic status of the recently described *Rhodobacter* species, *R. thermarum* (Khan et al., 2019), *R. flagellatus* (Xian et al., 2020), *R. sediminicola* (Suresh et al., 2020), and *R. tardus* (Sheu et al., 2020), was also not included in the early reclassification of *Rhodobacter* due to the nearly parallel time of description, causing confusion in the classification of the genus *Rhodobacter*.

The genus *Xinfangfangia*, described by Hu et al. (2018), is closely related to the genera of *Rhodobacter* and *Tabrizicola* within the family Rhodobacteraceae (Hu et al., 2018). It contains only two species with validated names, namely, *Xinfangfangia soli* and *Xinfangfangia humi* (Kämpfer et al., 2019). The genus *Pseudogemmobacter* described by Suman et al. (2019) contains only one species with validly published names, namely, *Pseudogemmobacter bohemicus.* While *X. humi* and *P. bohemicus*, which were proposed almost simultaneously, share a high 16S rRNA gene sequence similarity of 99.2%. Therefore, the relationship of *P. bohemicus* and *X. Humi* needs to be studied.

Along with the advances in whole-genome sequencing technologies, several methods for taxonomic classification at the species and genus levels have been developed. The new standards for species recognition are developed using digital whole-genome comparisons, such as average nucleotide identities (ANIs) (Konstantinidis and Tiedje, 2005) and genome-to-genome-distance calculations (GGDCs) (Richter and Rosselló-Móra, 2009; Meier-Kolthoff et al., 2013). The average amino acid identity (AAI) (Luo et al., 2014; Rodriguez-R and Konstantinidis, 2014) and percentage of conserved proteins (POCPs) (Qin et al., 2014), which are methods of measuring amino acid-level genomic similarity between protein-coding regions, have been used in the delineation of prokaryotic organisms at the genus level. Furthermore, the phylogenetic analysis based on the whole-genome sequence has been recently encouraged for the taxonomy of prokaryotes owing to its robustness and repeatability (Chun et al., 2018). Nowadays, along with those methods, the reclassification of prokaryotes at class (Hördt et al., 2020), order (Orata et al., 2018), family (Liang et al., 2021), and genus (Suresh et al., 2019) levels has been done.

In this study, phylogenetic analysis based on the 16S rRNA gene, *gyr*B gene, and genomes sequence, as well as ANI, AAI, and POCP, was used to confirm the taxonomic relationship of the novel strain and its closely related members (e.g., members of the genus *Xinfangfangia* and *Rhodobacter*) in the family Rhodobacteraceae.

## Materials and Methods

### Strain and Culture Conditions

Strain D13-10-4-6^T^ was isolated from the bark sample of *Populus* × *euramericana* collected from Heze, Shandong Province, China (34° 82′N, 115° 46′E) as previously described (Li et al., 2015). In brief, the bark samples were sterilized for 30 s with 70% ethanol, and then exposed to 4% (v/v) sodium hypochlorite for 3 min. After rinsing in sterile water three times, the samples with 2 ml sterile water were transferred to sterile mortar and ground with a pestle, respectively. The obtained solution was then shaken for 30 min at 30°C. The suspension with a dilution series was spread on tryptic soy agar (TSA, Difco). After 2 days of incubation on TSA plates at 30°C, single colonies were selected and cultured on a new plate, and were then preserved at -80°C with a supplement of 20% (v/v) glycerol.

### Genome Sequencing

The genomes of the strains D13-10-4-6^T^ and *X. soli* ZQBW^T^ were sequenced by Illumina NovaSeq PE150 (Novogene, Co., Ltd., Beijing, China). Low-quality reads in the raw data were filtered by readfq (version 10), then the genome assembly with high-quality reads was performed using SOAPdenovo (version 2.04) (Li et al., 2008; Li et al., 2010), SPAdes (Bankevich et al., 2012), ABySS (Simpson et al., 2009), and then the results were integrated with CISA (Lin and Liao, 2013). The gap of the genome assembly was filled using gapclose (version 1.12).

### Phylogenetic Analysis

The 16S rRNA gene of strain D13-10-4-6^T^ was amplified by the primers 27F/1492R (Lane, 1991). The similarity of the 16S rRNA gene sequence between the strain D13-10-4-6^T^ and the validly published bacterial species was determined using EzBio-Cloud’s identify service^3^ (Yoon et al., 2017). The 16S rRNA gene sequence of the related strains was obtained from GenBank for the phylogenetic analysis. After multiple sequence alignment with Clustal W, the phylogenetic analysis was carried out using MEGA X by the neighbor-joining, maximum-likelihood, and maximum-parsimony methods (Kumar et al., 2018). *Aquidulcibacter paucihalophilus* TH1-2^T^ was used as an outgroup. The phylogenetic trees were evaluated by 1,000 bootstrap resamplings.

The *gyr*B gene sequences of the strain D13-10-4-6^T^ were obtained from its genomic sequences according to Altschul et al. (1990), and a 1,050 bp sequence was obtained. The *gyr*B gene sequences of the related strains were obtained from GenBank or their genome sequences. The phylogenetic trees based on the *gyr*B gene sequence were constructed using the maximum-likelihood, neighbor-joining, and maximum-parsimony methods as a description of 16S rRNA gene phylogenetic analysis.

Concatenated protein tree has a higher recognition than single phylogenetic marker gene tree (e.g., 16S rRNA and *gyr*B) for bacterial taxonomy (Ciccarelli et al., 2006; Thiergart et al., 2014), and has been widely used in solving bacterial taxonomy (Hördt et al., 2020; Xu et al., 2021). The genome sequences of the strain D13-10-4-6^T^ and its related strains retrieved from GenBank (Supplementary Table 1) were used to construct the phylogenetic tree. A concatenated alignment of 120 ubiquitous single-copy proteins of the related strains was performed by GTDB-Tk v1.5.1^4^ using the classify_wf command (Chaumeil et al., 2020). The alignment file was used to construct the maximum-likelihood tree with IQ-TREE 2.1.4-beta (Minh et al., 2020), and the best model was automatically selected by ModerFinder for the ML tree. The tree was visualized and edited with iTOL (Letunic and Bork, 2021).

### Phylogenomic Metric Calculations

Average nucleotide identity (ANI is a measure of similarity between two genomic sequences, which is a useful tool to differentiate bacterial species in common with DNA-DNA hybridization (DDH) (Goris et al., 2007; Richter and Rosselló-Móra, 2009). The ANI values among the novel strain D13-10-4-6^T^ and its closely related reference strains (*P. bohemicus* Cd-10^T^, *X. humi* IMT-291^T^) were determined using OrthoANI (Lee et al., 2016). The GGDC^5^ was used to calculate the dDDH values among the novel and its closely related reference strains (Meier-Kolthoff et al., 2013). The analysis of the average AAI and POCP among the strains in this work was carried out with CompareM^6^ and a Python script (POCP)^7^ (Xu et al., 2020), respectively. The pan-genome analysis was carried out with BPGA (Chaudhari et al., 2016) with default parameters.

### Chemotaxonomic Characterization

The strain D13-10-4-6^T^ was shaken for 48 h in a tryptic soy broth (TSB; Difco) at 30°C, then collected by centrifuging at 10,000 rpm for 4 min. The harvested cells were freeze-dried and used to analyze the polar lipid and respiratory quinone. Polar lipids were analyzed by two-dimensional thin-layer chromatography as described by Minnikin et al. (1984). Isoprenoid quinones were extracted from the strain D13-10-4-6^T^ as reported by Collins et al. (1977), analyzed by high-performance liquid chromatography (Groth et al., 1997; Du et al., 2013), and confirmed by liquid chromatography/mass spectrometry. After culturing for 2 days in TSB at 30°C, the cells were harvested at exponential phase and used for cellular fatty acids. Cellular fatty acids were extracted as reported by Kuykendall et al. (1988), analyzed using the Sherlock Microbial Identification System (Sasser, 1990).

### Phenotypic Characterization

Growth conditions of the strain D13-10-4-6^T^ were determined at different temperature, pH, and salinity levels according to the method described by Li et al. (2016). The growth temperature was set at 4, 10, 15, 20, 25, 28, 30, 37, 41, and 45°C. The pH values for growth were adjusted to various pH values (pH 4.0–11.0, at intervals of 1.0 pH unit) by the buffers (Delory and King, 1945; Gomori, 1955) citrate and Na_2_HPO_4_ buffer (pH 4.0–5.0), phosphate buffer (pH 6.0–7.0), Tris buffer (pH 8.0–9.0), and Na_2_HPO_4_/NaOH (pH 10.0–11.0). The salinity was determined in the range of 0–9% (w/v, intervals of 1%). Gram staining was performed according to the method described by Jenkins et al. (2003). To examine the anaerobic growth, the strain was incubated on TSA plates at 30°C for 1 week in an anaerobic jar (Li et al., 2016). The activities of catalase and oxidase were determined by the methods described by Smibert and Krieg (1994). Enzymatic activity, carbon source utilization, and acid production were performed by API ZYM, API 20 NE, and API 50 CH (bioMérieux) according to the manufacturer’s instructions, respectively.

## Results and Discussion

### Genome Information

The genome of strains D13-10-4-6^T^ and *X. soli* ZQBW^T^ were sequenced and analyzed. In total, 63 contigs with a total sequence length of 4,683,906 bp for the strain *X. soli* ZQBW^T^ were obtained, which was predicted to have 4,455 protein-coding genes, 47 tRNA genes, 3 rRNA genes, and 3 other RNA genes. The DNA G + C contents was 67.6%. The strain D13-10-4-6^T^ genome produced 66 contigs with a total sequence length of 4,605,234 bp, which was predicted to have 4,206 protein-coding genes, 45 tRNA genes, 3 rRNA genes, and 3 other RNA genes. The DNA G + C content of the strain D13-10-4-6^T^ was 62.9%, which was similar to *P. bohemicus* Cd-10^T^ (63.2%).

### Phylogenetic Analyses

**FIGURE 1:**
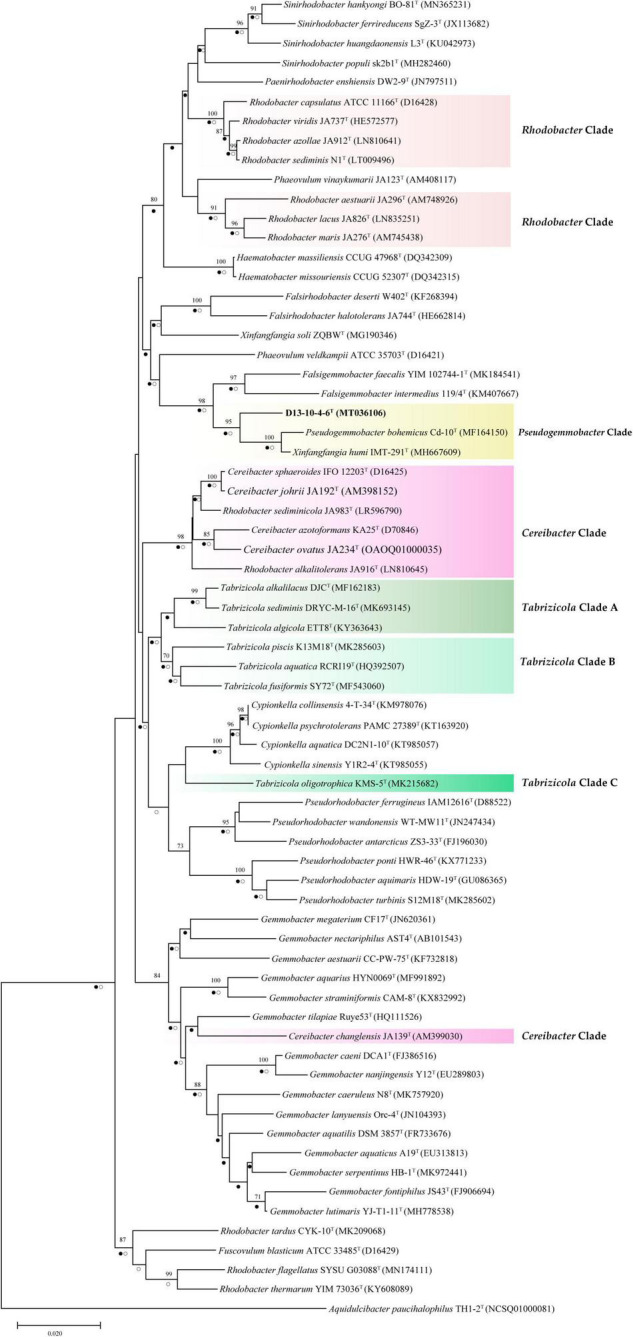
Neighbor-joining tree showing phylogenetic relationships among strain D13-10-4-6^T^ and reference strains based on 16S rRNA gene sequences. *Aquidulcibacter paucihalophilus* TH1-2^T^ was used as an outgroup. Only bootstrap values over 70% (based on 1,000 resamplings) are shown. The scale bar corresponds to 0.01 substitutions per nucleotide site. Filled circles indicate branches recovered by maximum-likelihood method and open circles at branches recovered by the maximum-parsimony method.

**FIGURE 2:**
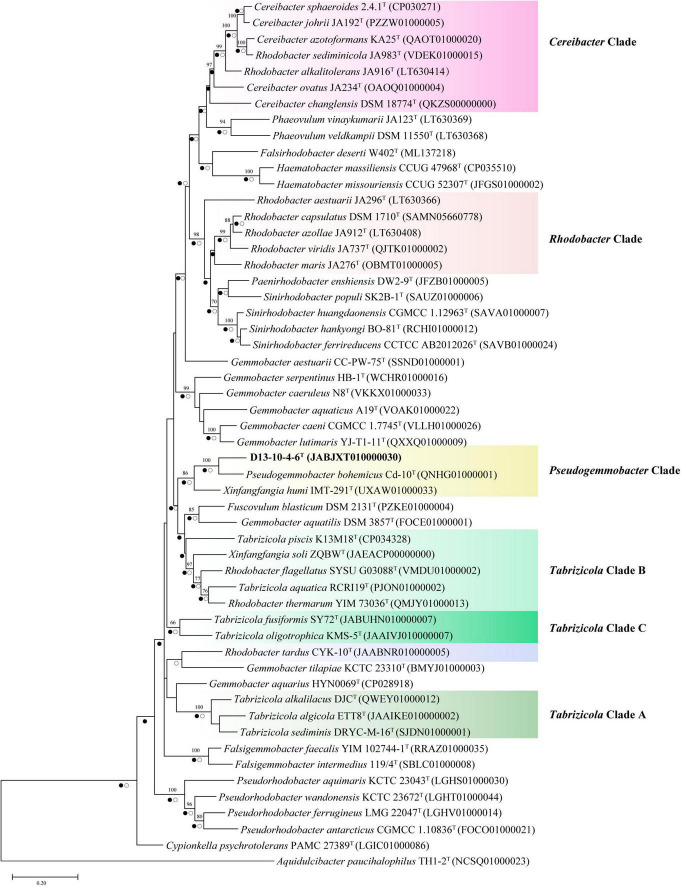
Neighbor-joining phylogenetic trees based on partial *gyr*B gene sequences showing the position of strain D13-10-4-6^T^ and reference strains. Bootstrap values over 70% (expressed as percentages of 1,000 replications) are shown. The scale bar corresponds to 0.05 substitutions per nucleotide site. Filled circles indicate branches recovered by maximum-likelihood method and open circles at branches recovered by the maximum-parsimony method.

**FIGURE 3:**
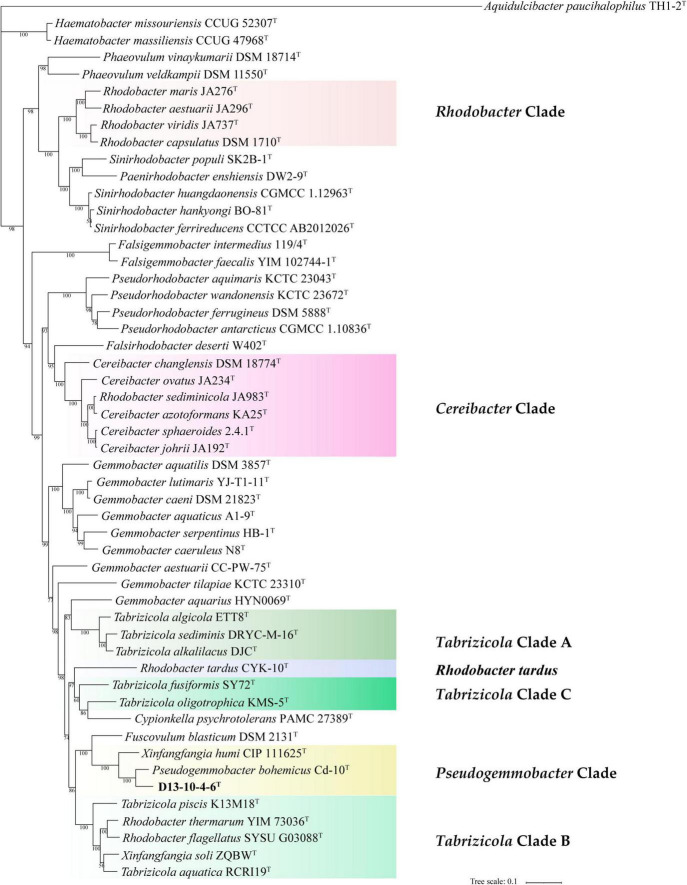
Phylogenetic tree among strain D13-10-4-6T and reference strains based on a concatenated alignment of 120 ubiquitous single-copy proteins. Aquidulcibacter paucihalophilus TH1-2T was used as an outgroup. The scale bar corresponds to 0.1 substitutions per amino acid position.

In this study, we have constructed phylogenetic trees based on the 16S rRNA gene, *gyr*B gene, and concatenated proteins (Figures 1–3) for representative members of the family Rhodobacteraceae encompassing 15 genera. The main groups clustering with the members of *Rhodobacteraceae* in 16S rRNA gene-based tree, *gyr*B gene-based tree, and concatenated proteins-based tree are almost consistent. The strains D13-10-4-6^T^, *P. bohemicus* Cd-10^T^, and *X. humi* IMT-291^T^ form one monophyletic group to in turn form *Pseudogemmobacter* clade with strong bootstrap support in all three phylogenetic trees (Figures 1–3), which is far removed from the branch of *X. soli* (the type species of the genus *Xinfangfangia)*. *X. humi* IMT-291^T^ forms a distinct branch from the strains D13-10-4-6^T^ and *P. bohemicus* Cd-10^T^ in the *Pseudogemmobacter* clade. The results suggested that *X. humi* IMT-291^T^ should be a species belonging to the genus *Pseudogemmobacter*, although *P. bohemicus* Cd-10^T^ and *X. humi* IMT-291^T^ were published almost simultaneously and shared 99.26% 16S rRNA gene sequence similarity with each other. The strain D13-10-4-6^T^ forms a distinct branch from *P. bohemicus* Cd-10^T^ and *X. humi* IMT-291^T^ in all phylogenetic trees, and it has the highest 16S rRNA gene sequence similarity to *P. bohemicus* Cd-10^T^ (97.6%) and *X. humi* IMT-291^T^ (97.4%), and shares a less than 97% sequence similarity with all other validly published species. The results indicate that the strain D13-10-4-6^T^ should belong to a novel species of the genus *Pseudogemmobacter.*

Several genera are non-monophyletic, such as *Rhodobacter*, *Tabrizicola*, and *Xinfangfangia*. In most of the cases, the 16S rRNA gene-based tree shows its low discriminatory power. For instance, the species of the genus *Tabrizicola* are divided into two branches in the 16S rRNA gene-based tree, but they are clustered into three distinct branches, not least *T. aquatica* RCRI19^T^ (type species of the genus) and *T. piscis* K13M18^T^ are grouped together with *R. thermarum* YIM 73036^T^, *R. flagellatus* SYSU G03088^T^, and *X. soli* ZQBW^T^ in trees based on *gyr*B gene and concatenated proteins tree with strong support.

It can be seen from the trees based on the 16S rRNA gene, *gyr*B gene, and concatenated proteins that *Rhodobacter* and *Tabrizicola*a are two closely related non-monophyletic genera. The members of the genus *Tabrizicola* are observed in three clades, clades A, B, and C, which are labeled in Figures 1–3. Clade A, formed by *Tabrizicola alkalilacus* DJC^T^, *Tabrizicola sediminis* DRYC-M-16^T^, and *Tabrizicola algicola* ETT8^T^, is next to the *Gemmobacter* clade and is far removed from two other *Tabrizicola* clades with a strong support in 16S rRNA gene-based, *gyr*B gene-based, and concatenated proteins-based trees. These results suggest that clade A should belong to a novel genus of the family Rhodobacteraceae. *Tabrizicola* clade B, grouped by *T. aquatica* RCRI19^T^ (type species of the genus), *T. piscis* K13M18^T^, *R. thermarum* YIM 73036^T^, *R. flagellatus* SYSU G03088^T^, and *X. soli* ZQBW^T^, is a monophyletic cluster found in trees based on the *gyr*B gene and concatenated proteins with a strong support, indicating that *R. thermarum* YIM 73036^T^, *R. flagellatus* SYSU G03088^T^, and *X. soli* ZQBW^T^ should be transferred to the genus *Tabrizicola. Tabrizicola fusiformis* SY72^T^, located in clade B in the 16S rRNA gene-based tree, is clustered together with *Tabrizicola oligotrophica* KMS-5^T^ to form clade C in both trees based on the *gyr*B gene and concatenated proteins, demonstrating that *T. fusiformis* SY72^T^ and *T. oligotrophica* KMS-5^T^ may be a novel genus of the family Rhodobacteraceae.

In the 16S rRNA gene-based tree, *Cereibacter* clade include *R. alkalitolerans* JA916^T^, *R. sediminicola* JA983^T^), and members of the genus *Cereibacter*, except for *Cereibacter changlensis* JA139^T^ (the type species). The species *C. changlensis* JA139^T^ is observed in the *Gemmobacter* clade, which is similar to the results reported by Suresh et al. (2015). While *C. changlensis* JA139^T^ is grouped in the *Cereibacter* clade and located at the edge of the clade in trees inferred from the *gyr*B gene and concatenated proteins, indicating that it should belong to the genus *Cereibacter*, which is consistent to the results described by Suresh et al. (2015). *R. sediminicola* JA983^T^ is clustered in the *Cereibacter* clade in all the trees based on the 16S rRNA gene, *gyr*B gene, and concatenated proteins, indicating that they should be transferred to the genus *Cereibacter*.

The genus *Rhodobacter* proposed by Imhoff et al. (1984) contains 13 species with validated names according to the LSPN. In trees based on the 16S rRNA gene, gyrB gene, and concatenated protein, *R. tardus* CYK-10^T^ forms one distinct branch from other clades, suggesting that it should belong to a novel genus of the family Rhodobacteraceae. Three members (*R. sediminicola* JA983^T^, *R. thermarum* YIM73036^T^, *R. flagellatus* SYSU G03088^T^) are clustered into the *Tabrizicola* clade and *Cereibacter* clade, respectively. The other eight members of the genus *Rhodobacter* are temporarily classified into the genus *Rhodobacter* because of the absence of genome sequence of *R. azollae*, *R. lacus*, *R. alkalitolerans*, and *R. sediminis*, although they are grouped into two clusters in the 16S rRNA gene tree.

### Genomic, Chemotaxonomic, and Physiological Analysis of the Novel Strain

The ANI values between the strain D13-10-4-6^T^ and its three closely related strains range from 74.4 to 81.2%, which are lower than the recommended ANI species boundary cutoff value (95–96%). The dDDH values between the strain D13-10-4-6^T^ and its closely related strains are 19.7–24.3%, lower than the threshold for species (70%). Those data indicate that the strain D13-10-4-6^T^ should belong to a novel species of the genus *Pseudogemmobacter*. Besides, *P. bohemicus* Cd-10^T^ and *X. humi* IMT-291^T^ share a 99.26% 16S rRNA gene sequence similarity, while their ANI and dDDH values are 79.1 and 22.1%, respectively (Table 1), which are lower than the species boundary cutoff values. Therefore, *P. bohemicus* Cd-10^T^ and *X. humi* IMT-291^T^ should belong to a different species of the genus *Pseudogemmobacter*.

**TABLE 1:** Average nucleotide identity (ANI), digital DNA–DNA hybridization (dDDH) values among D13-10-4-6^T^, *P. bohemicus* Cd-10^T^, *X. humi* IMT-291^T^, and *Xinfangfangia soli* ZQBW^T^.

Strain	D13-10-4-6^T^		Cd-10^T^		IMT-291^T^	
	ANI	dDDH	ANI	dDDH	ANI	dDDH
strain D13-10-4-6^T^	100					
*Pseudogemmobacter bohemicus* Cd-10^T^	81.2	24.3	100			
*Xinfangfangia humi* IMT-291^T^	78.3	22.0	79.1	22.1	100	
*Xinfangfangia soli* ZQBW^T^	74.4	19.7	74.7	19.4	76.7	20.3

The polar lipids of the strain D13-10-4-6^T^ are phosphatidy- lmonomethylethanolamine (PME), diphosphatidylglycerol (DPG), phosphatidylethanolamine (PE), phosphatidylglycerol (PG), phosphatidylcholine (PC), an unidentified phospholipid (PL), and six unidentified lipids (L) (Supplementary Figure 4). The presence of PE in the strain D13-10-4-6^T^ is a useful characteristic to distinguish it from *P. bohemicus* and *X. soli*. The presence of DPG and absence of PC in the strain D13-10-4-6^T^ are important characteristics to differentiate it from *X. humi*. The respiratory quinones detected in the strain D13-10-4-6^T^ are Q-10 (91.3%) and Q-9 (8.7%), which are similar to *P. bohemicus* Cd-10^T^ and *X. humi* IMT-291^T^. *X.* soli contains the only respiratory quinone of Q-10, which is different from the strains D13-10-4-6^T^, *P. bohemicus* Cd-10^T^, and *X. humi* IMT-291^T^. The phenotypic characterization of the strain D13-10-4-6^T^ is listed in Table 2 and in the species description.

**TABLE 2:** Differential characteristics of strain D13-10-4-6^T^ and closely related reference strains.

Characteristic	1	2	3	4
Cell shape	Ovoid to rod-shaped	Ovoid to rod-shaped	Rod-shaped	Rod-shaped
Colour of colonies	Creamy white	Creamy white to	Beige	Light yellow
		Beige		
Optimum pH	7.0–8.0	7.0–8.0	5.5–6.5	7
Optimum temperature (°C)	25–30	28	20–28	30
Growth in max NaCl (%, w/v)	4	1	2	2
Reduction of nitrate, indole production	–	+	–	–
Utilization of:				
D-Glucose, D-mannose, D-mannitol	+	+	–	–
L-Arabinose	+	–	+	–
D-Maltose	–	+	–	–
L-Rhamnose, *N*-acetyl-glucosamine	+	–	–	–
Enzyme activities:				
Lipase (C14)	W	+	–	–
Valine arylamidase	W	+	–	W
Cystine arylamidase	–	+	–	W
α-Chymotrypsin	–	+	–	–
α-Glucosidase	+	–	–	+
*N*-acetyl-β-glucosaminidase	+	–	–	–
Trypsin	–	+	–	–
Hydrolysis from:				
Aesculin	+	–	–	–
Gelatin	–	–	+	–
Predominant polar lipids	PME, DPG, PE, PG	PME, PG, DPG, PC,	PE, PME, PG, PC,	PC, PG, PME
G + C content (%)	62.9	63.2	66.5	67.0

*1, strain D13-10-4-6^T^; 2, Pseudogemmobacter bohemicus Cd-10^T^ (data from Suman et al., 2019); 3, Xinfangfangia humi IMT-291^T^ (data from Kämpfer et al., 2019); 4, Xinfangfangia soli ZQBW^T^ (data from this study). +, positive; -, negative; W, weakly positive.*

The predominant fatty acids of the strain D13-10-4-6^T^ are C_18:1_ ω7*c* (81.1%), C_16:0_ (5.4%), and C_18:0_ (4.1%). The detailed and differential fatty acids data of strain D13-10-4-6^T^ and its related species are listed in Table 3. The percentage of C_18:1_ω7*c* in the novel strain can be used to distinguish it from *P. bohemicus* Cd-10^T^ and *X. humi* IMT-291^T^. The absence of 11-methyl C_18:1_ ω7*c* in the strain D13-10-4-6^T^ is a useful characteristic to differentiate it from *X. soli* ZQBW^T^.

**TABLE 3:** Cellular fatty acid profiles of strain D13-10-4-6^T^ and closely related type strains.

Fatty acid	1	2	3	4
C_18:0_	4.1	26.3	2.5	3.1
C_16:0_	5.4	19.9	10.5	3.5
C_16:1_ ω7*c*	1.5	2.9	NA	–
C_10:0_ 3-OH	2.4	NA	2.8	0.9
C_18:0_ 3-OH	2.6	NA	NA	1.4
11-methyl C_18:1_ ω7*c*	–	NA	22.7	2.3
C_18:1_ω7*c*	81.1	50.3	58.8	85.2

*1, strain D13-10-4-6^T^; 2, Pseudogemmobacter bohemicus Cd-10^T^ (data from Suman et al., 2019); 3, Xinfangfangia humi IMT-291^T^ (data from Kämpfer et al., 2019); 4, Xinfangfangia soli ZQBW^T^ (data from this study). NA, not available; -, not detected.*

### Phylogenomic Metric Analysis

Average AAI is one of the well-established methods to separate prokaryotic genera (Luo et al., 2014; Rodriguez-R and Konstantinidis, 2014). It is proposed to be 65% AAI value for genera delineation of Bacteria and Archaea (Konstantinidis et al., 2017). However, the category thresholds of AAI for genus delineation are variable in many genera. For example, the value of 70% AAI is used to separate the genus *Geomonas* from the other genera of the family Geobacteraceae (Xu et al., 2020), and a range of 64.6–77.0% AAI is used to delineate different genera of the family Geobacteraceae (Xu et al., 2019). In this study, the values of AAI among 52 type strains from 15 related genera of the family Rhodobacteraceae have been determined and are listed in Table 4 and Supplementary Table 2. It can be seen from Table 4 that a gradient of 63.5–75.3% and 74.2–98.7% AAI values is found among the different clade (genera) and in the same clade of the family Rhodobacteraceae. Those data are consistent to the results of phylogeny based on concatenated proteins.

**TABLE 4:** Average amino acid identity (AAI) values and percentage of conserved protein (POCP) values of all strains for the intragenus and intergeneric comparisons.

Organism	AAI (%)		POCP (%)	
	Intragenus	Intergeneric	Intragenus	Intergeneric
*Pseudogemmobacter* clade	77.4–81.8	63.8–73.6	61.0–62.9	41.7–59.0
*Cereibacter* clade	75.8–98.7	65.2–72.7	60.9–88.6	44.6–62.7
*Rhodobacter* clade	79.5–90.1	64.2–75.3	70.4–79.3	41.9–65.5
*Rhodobacter tardus*	100	63.5–70.9	100	42.8–61.9
*Tabrizicola* clade A	84.6–87.0	63.9–73.2	70.4–73.3	45.6–65.7
*Tabrizicola* clade B	79.5–86.7	64.2–74.4	65.9–85.1	45.2–68.1
*Tabrizicola* clade C	76.18	65.0–74.0	67.4	45.9–67.8
*Gemmobacter*	69.2–93.0	64.7–73.26	51.0–77.7	42.2–67.8
*Cypionkella*	100	63.8–74.0	100	42.7–63.4
*Falsigemmobacter*	90.2	63.8–66.6	79.7	42.7–52.2
*Falsirhodobacter*	100	64.6–69.6	100	41.7–51.5
*Fuscovulum*	100	65.0–74.4	100	48.2–68.1
*Haematobacter*	92.68	63.8-68.1	81.8	44.0–53.2
*Paenirhodobacter*	100	63.8–74.4	100	45.7–63.8
*Phaeovulum*	74.2	64.7–72.4	69.2	45.2–65.5
*Pseudorhodobacter* clade	78.3–83.5	63.9–71.5	61.9–73.2	44.3–67.2
*Sinirhodobacter*	74.4–96.1	63.5–75.3	56.5–88.3	44.3–63.8

*Pseudogemmobacter clades include strain D13-10-4-6^T^, Pseudogemmobacter bohemicus Cd-10^T^, and Xinfangfangia humi IMT-291^T^. Cereibacter clades include Rhodobacter sediminicola JA983^T^ and members of the genus Cereibacter. Rhodobacter clades include Rhodobacter maris JA276^T^, Rhodobacter aestuarii JA296^T^, Rhodobacter capsulatus DSM 1710^T^, and Rhodobacterviridis JA737^T^. Tabrizicola clade A includes Tabrizicola alkalilacus DJC^T^, Tabrizicola sediminis DRYC-M-16^T^, and Tabrizicola algicola ETT8^T^. Tabrizicola clade B includes Xinfangfangia soli ZQBW^T^, Rhodobacter flagellatus SYSU G03088^T^, Rhodobacter thermarum YIM 73036^T^, Tabrizicola aquatic RCRI19^T^, and Tabrizicola piscis K13M18^T^, Tabrizicola clade C includes Tabrizicola fusiformis SY72^T^ and Tabrizicola oligotrophica KMS-5^T^.*

**FIGURE 4:**
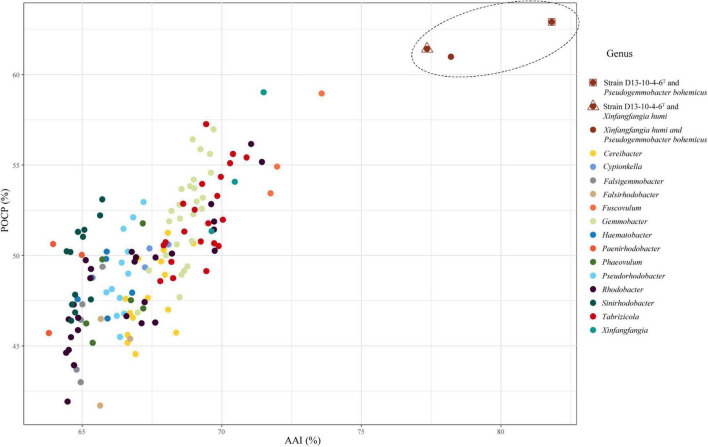
The relationship of AAI and POCP between strain D13-10-4-6^T^, *P. bohemicus* Cd-10^T^, *X. humi* IMT-291^T^ and the related strains in the family *Rhodobacteraceae.* The dots inside the dashed line represent the values between strain D13-10-4-6^T^, *P. bohemicus* Cd-10^T^ and *X. humi* IMT-291^T^, and those outside represent the values between the three strains and strains in the related genera of the family *Rhodobacteraceae.* A total of 52 genomes were included in this analysis.

The *Pseudogemmobacter* clade, including strains D13-10-4-6^T^, *P. bohemicus* Cd-10^T^ and *X. humi* IMT-291^T^, has 77.4–81.8% AAI values among each other and shows 63.8–73.6% AAI values among the members of other clades in this study (Table 4 and Figure 4), which is consistent to the results of phylogeny based on the 16S rRNA gene, *gyr*B gene, and concatenated proteins (Figures 1–3). The *Cereibacter* clade, including *R. sediminicola* JA983^T^ and members of the genus *Cereibacter*, has 75.8–98.7% AAI values among the members of the clade and 65.2–72.7% AAI values among the members from the other clades in this work, indicating that *R. sediminicola* should be transferred to the genus *Cereibacter* (Table 4 and Supplementary Figure 1). Similarly, the AAI values within the *Tabrizicola* clade A and *Tabrizicola* clade B can also distinguish them from the other strains (Table 4 and Supplementary Figures 2, 3).

Percentage of conserved protein is another method for genus delineation of prokaryote, and the value for genera delineation of POCP is proposed to be 50% (Qin et al., 2014). While the thresholds of POCP for genus delineation are also variable in many genera. The value of 65% POCP was used to separate the genus *Geomonas* from the other genus of the family Geobacteraceae (Xu et al., 2020), and most of the POCP values within the *Roseobacter* group comparisons were greater than 50% of the family Rhodobacteraceae (Wirth and Whitman, 2018). In this work, we also calculated the POCP values among the 52 type strains of 15 related genera of the family Rhodobacteraceae (Supplementary Table 2). The results show that a gradient of 41.7–68.1% POCP, except the values between, was found among the different clade (genera) of the family Rhodobacteraceae, and 56.5–88.6% among the species of the same clade. Therefore, it is hard to use the same thresholds for genus delineation because they show a broad range of values from both intragenus and intergeneric. But for several clades, it is useful to distinguish one group from the others, for instance, members of the *Pseudogemmobacter clade* show the values of POCP from 61.0 to 62.9% among each other and have 41.7–59% POCP values among members from the other clades (Table 4 and Figure 4). The same goes for the *Cereibacter* clade and *Tabrizicola* clade A (Table 4 and Supplementary Figures 1, 2).

The POCP values in this study are not always consistent to the phylogenetic analysis, as exemplified by the *Tabrizicola* clade B. Members of the *Tabrizicola* clade B show 45.2–68.1% POCP values among each other, and 65.9–85.1% POCP values among the members of other clades, respectively (Table 4). The POCP values can distinguish *Tabrizicola* clade B from other related members except for *T. oligotrophica* KMS-5^T^, *Fuscovulum blasticum* DSM 2131^T^, and *Gemmobacter aestuarii* CC-PW-75^T^ (Supplementary Figure 3). The POCP values between *X. soli* ZQBW^T^ (belonging to *Tabrizicola* clade B) and *T. oligotrophica* KMS-5^T^, *F. blasticum* DSM 2131^T^, and *G. aestuarii* CC-PW-75^T^ were slightly higher than those between *X. soli* ZQBW^T^ and other members in *Tabrizicola* clade B (Supplementary Figure 3). Due to *X. soli* ZQBW^T^ forming a stable clade within *Tabrizicola* clade B and forming a distinct clade with *T. oligotrophica* KMS-5^T^, *F. blasticum* DSM 2131^T^, and *G. aestuarii* CC-PW-75^T^, we analyzed the pan-genome of *Tabrizicola* clade B in a supplementary analysis.

The pan-genome analysis was used in the classification of bacteria (Awan et al., 2018; Suresh et al., 2019). The amount of core genes was sensitive to heterogeneous in the core- and pan-genome analysis (Inglin et al., 2018; Suresh et al., 2019). The core gene numbers within were *Tabrizicola* clade B were considerably higher than those within the *Tabrizicola* clade B and *T. oligotrophica* KMS-5^T^, *F. blasticum* DSM 2131^T^, and *G. aestuarii* CC-PW-75^T^ (Table 5), indicating that the relationships of *Tabrizicola* clade B and the three species were heterogeneous and the *Tabrizicola* clade B should belong to a genus different from the three species. The pan-genome analysis reinforces the results of *gyr*B and concatenated protein phylogenetic trees. In conclusion, *Tabrizicola* clade B should belong to the same genus.

**TABLE 5:** Pan-genomic analysis of *Tabrizicola* clade B and the related strains.

Clade	Organism name	No. of core genes	No. of other genes
All eight strains	*Rhodobacter flagellatus* SYSU G03088^T^	1,656	1,892
	*Rhodobacter thermarum* YIM 73036^T^	1,656	1,873
	*Tabrizicola aquatica* RCRI19^T^	1,656	2,112
	*Tabrizicola piscis* K13M18^T^	1,656	2,522
	*Xinfangfangia soli* ZQBW^T^	1,656	2,877
	*Tabrizicola oligotrophica* KMS-5^T^	1,656	2,087
	*Fuscovulum blasticum* DSM 2131^T^	1,656	1,896
	*Gemmobacter estuarii* CC-PW-75^T^	1,656	1,933
*Tabrizicola* clade B	*Rhodobacter flagellatus* SYSU G03088^T^	2,144	1,442
	*Rhodobacter thermarum* YIM 73036^T^	2,144	1,414
	*Tabrizicola aquatica* RCRI19^T^	2,144	1,651
	*Tabrizicola piscis* K13M18^T^	2,144	2,126
	*Xinfangfangia soli* ZQBW^T^	2,144	2,571

## Conclusion

Phylogenetic trees based on the 16S rRNA gene, *gyr*B gene, and concatenated alignment of 120 ubiquitous single-copy proteins were constructed to clarify the relationship of the members from the 15 closely related genera within the family Rhodobacteraceae. The AAI, POCP, and ANI analyses as well as chemotaxonomic and physiological tests were also performed and used as supplementary evidence. On the basis of the data obtained, the taxonomic proposals were (1) reclassification of *R. tardus* as the type species of a novel genus, *Stagnihabitans* gen. nov., as *Stagnihabitans tardus* comb. nov.; (2) reclassification of *T. alkalilacus*, *Tabrizicola sediminis*, and *Tabrizicola algicola* into a novel genus, *Pseudotabrizicola* gen. nov., as *Pseudotabrizicola alkalilacus* comb. nov., *Pseudotabrizicola sediminis* comb. nov., *Pseudotabrizicola algicola* comb. nov.; (3) reclassification of *Rhodobacter sediminicola* into the genus *Cereibacter* as *Cereibacter sediminicola* comb. nov.; (4) reclassification of *Rhodobacter flagellatus*, *Rhodobacter thermarum*, and *X. soli* into the genus *Tabrizicola* as *Tabrizicola flagellatus* comb. nov., *Tabrizicola thermarum* comb. nov., and *Tabrizicola soli* comb. nov.; (5) reclassification of *X. humi* into the genus *Pseudogemmobacter* as *Pseudogemmobacter humicola* comb. nov.; and (6) classification of strain D13-10-4-6^T^ as a novel species of the genus *Pseudogemmobacter*, for which the name *Pseudogemmobacter hezensis* sp. nov. is proposed; the type strain is D13-10-4-6^T^ (= CFCC 12033^T^ = KCTC 82215^T^).

### Description of *Pseudogemmobacter hezensis* Sp. nov.

*Pseudogemmobacter hezensis* (he.zen’sis. N.L. masc./fem. adj. *hezensis*, of Heze, a city in Shandong Province, China, where the organism was isolated).

Cells are Gram-stain-negative, aerobic, non-motile, catalase- and oxidase-positive, ovoid to rod-shaped, 1.6–2.0 μm in length and 0.8–1.0 μm in width. Colonies are creamy white, circular, smooth, with entire margins after incubation for 2 days at 28°C on TSA. The strain can grow at 15–37°C (optimum, 25–30°C), at pH 6–10 (optimum, pH 7–8). Growth occurs at a concentration of 0–4% (w/v) NaCl. It is positive for the activity of alkaline phosphatase, esterase lipase (C8), esterase (C4), leucine arylamidase, valine arylamidase, acid phosphatase, naphthol-AS-BI-phosphohydrolase, α-glucosidase, *N*-acetyl-β-glucosaminidase; weakly positive for lipase (C14), β-galactosidase; negative for cystine arylamidase, trypsin, α-chymotrypsin, α-galactosidase, β-glucuronidase, β-glucosidase, α-mannosidase, and α-fucosidase (API ZYM). It is negative for reduction of nitrate to nitrogen, reduction of nitrate to nitrite, indole production, gelatin hydrolysis, and the activity of urease, arginine dihydrolase; positive for the utilization of glucose, D-mannose, L-arabinose, D-mannitol, *N*-acetyl-glucosamine, malic acid (API 20 NE). Acid is produced from L-arabinose, D-xylose, D-galactose, D-fructose, L-rhamnose, D-lyxose, D-fucose, L-fucose; weakly positive for erythritol, D-arabinose, D-ribose, and L-sorbose (API 50 CH). The polar lipids are PME, DPG, PE, PG, PC, PL1, and six unidentified lipids (L). The respiratory quinones are Q-10 and Q-9. The predominant fatty acids are C_18:1_ω7*c*. The type strain is D13-10-4-6^T^ (= CFCC 12033^T^ = KCTC82215^T^), isolated from the bark samples of *Populus* × *euramericana* in Shandong Province, China. The DNA G + C content is 62.9%.

The GenBank/EMBL/DDBJ accession numbers for the 16S rRNA gene and genome sequences for strain D13-10-4-6^T^ is MT036106 and JABJXT000000000, respectively.

### Description of *Pseudogemmobacter humi* Comb. nov.

*Pseudogemmobacter humi* (hu’mi. L. gen. fem. n. humi, of/from soil, the isolation source of the type strain).

Basonym: *Xinfangfangia humi* (Kämpfer et al., 2019).

The description of *Pseudogemmobacter humi* is the same as that given for *X. humi* by Kämpfer et al. (2019). The type strain is IMT-291^T^ (= LMG 30636^T^ = CIP 111625^T^ = CCM 8858^T^).

### *Emended Description of the Genus* Pseudogemmobacter

The description as given by Suman et al. (2019) remains correct except that the species are positive or negative for catalase and nitrate reductase.

### Description of *C. sediminicola* Comb. nov.

*Cereibacter sediminicola* (se.di.mi.ni’co.la. L. neut. n. sedimen, sediment; L. masc./fem. suff. -cola, inhabitant, dweller; from L. masc./fem. n. incola, dweller; N.L. masc./fem. n. sediminicola, dweller of sediments).

Basonym: *Rhodobacter sediminicola* (Suresh et al., 2020).

The description of *C. sediminicola* is the same as that given for *R. sediminicola* by Suresh et al. (2020). The type strain is JA983^T^ (= KCTC 15782^T^ = NBRC 113843^T^).

### Description of *Stagnihabitans* Gen. nov.

*Stagnihabitans* (Sta.gni.ha’bi.tans. L. neut. n. *stagnum*, a small area of water, pond; L. pres. part. *Habitans*, an inhabitant; N.L. masc. n. *Stagnihabitans*, an inhabitant of pond water).

Cells are Gram-strain-negative, aerobic, non-motile, oxidase-positive, catalase-negative, ovoid to rod-shaped and divide by binary fission, sometimes forming chains. The predominant respiratory quinone is Q-10. The major cellular fatty acid is C_18:1_ω7*c*. PE, PG, and PC are the major polar lipids. The DNA G + C content is 66%. The member of the genus is separated from *Rhodobacter* based on the 16S rRNA, gyrB and concatenated protein phylogenetic trees, genome comparison. The type species is *S. tardus* comb. nov.

### Description of *S. tardus* Comb. nov.

*Stagnihabitans tardus* (tar’dus. L. masc.adj. *tardus*, slow, referring to the slow growth of the organism).

Basonym: *Rhodobacter tardus* (Sheu et al., 2020).

The description of *S. tardus* is the same as that given for *R. tardus* by Sheu et al. (2020). The type strain is CYK-10^T^ (= BCRC 81191^T^ = LMG 31336^T^).

### Description of *Pseudotabrizicola* Gen. nov.

*Pseudotabrizicola* (Pseu.do.ta.bri.zi.co.la. Gr. masc./fem. adj. pseudês, false; N.L. fem. n. *Tabrizicola*, a bacterial generic name; N.L. fem. n. *Pseudotabrizicola*, false Tabrizicola).

Cells are Gram-strain-negative, aerobic, non-motile, catalase- and oxidase-positive, rod-shaped. PG, DPG, PE, and PC are the major polar lipids. The predominant respiratory quinone is Q-10. The major cellular fatty acids are usually iso-C_18:0_, C_18:1_ ω7*c*, and/or C_18:1_ ω6*c*. The DNA G + C content is 62.9–64.4%. Members of the genus are separated from *Tabrizicola* based on the 16S rRNA, gyrB, and concatenated proteins phylogenetic trees, genome comparison. The type species is *P. sediminis* comb. nov.

### Description of *P. sediminis* Comb. nov.

*Pseudotabrizicola sediminis* (se.di’mi.nis. L. gen. net. n. *sediminis*, of a sediment).

Basonym: *Tabrizicola sediminis* (Liu et al., 2019).

The description of *P. sediminis* is the same as that given for T*abrizicola sediminis* by Liu et al. (2019). The type strain is DRYC-M-16^T^ (= CGMCC 1.13881^T^ = KCTC 72105^T^).

### Description of *P. alkalilacus* Comb. nov.

*Pseudotabrizicola alkalilacus* (al.ka.li.la’cus. N.L. neut. n. *alkali* from Arabic article al, the; Arabic n. *qaliy*, ashes of saltwort, alkali; L. masc. n. *lacus*, a lake; N.L. gen. masc. n. *alkalilacus* of analkaline lake).

Basonym: *Tabrizicola alkalilacus* (Phurbu et al., 2019).

The description of *Pseudotabrizicola alkalilacus* is the same as that given for *T. alkalilacus* by Phurbu et al. (2019). The type strain is DJC^T^ (= CICC 24242^T^ = KCTC 62173^T^).

### Description of *P. algicola* Comb. nov.

*Pseudotabrizicola algicola* (al.gi’co.la. L. fem. n. *algae*, an alga; L. masc./fem. suff.*-cola*, dweller; from L. masc./fem. n. *incola* an inhabitant; N.L. masc./fem. n. *algicola* an inhabitant of algae).

Basonym: *Tabrizicola algicola* (Park et al., 2020).

The description of *P. algicola* is the same as that given for *Tabrizicola algicola* by Park et al. (2020). The type strain is ETT8^T^ (= KCTC 72206^T^ = JCM 31893^T^ = MCC 4339^T^).

### Description of *T. flagellatus* Comb. nov.

*Tabrizicola flagellatus* (fla.gel.la’tus. L. masc. part. adj. *flagellatus*, flagellated).

Basonym: *Rhodobacter flagellatus* (Xian et al., 2020).

The description of *T. flagellatus* is the same as that given for *Rhodobacter flagellatus* by Xian et al. (2020). The type strain is SYSU G03088^T^ (= CGMCC 1.16876^T^ = KCTC 72354^T^).

### Description of *T. thermarum* Comb. nov.

*Tabrizicola thermarum* (ther.ma’rum. L. gen. fem. pl. n. *thermarum*, of hot springs).

Basonym: *Rhodobacter thermarum* (Khan et al., 2019).

The description of *T. thermarum* is the same as that given for *Rhodobacter thermarum* by Khan et al. (2019). The type strain is YIM 73036^T^ (= KCTC 52712^T^ = CCTCC AB 2016298^T^).

### Description of *T. soli* Comb. nov.

*Tabrizicola soli* (so’li. L. neut. n. *soli* of soil, the source of the type strain).

Basonym: *Xinfangfangia soli* (Hu et al., 2018).

The description of *T. soli* is the same as that given for *X. soli* by Hu et al. (2018). The type strain is ZQBW^T^ (= KCTC 62102^T^ = CCTCC AB 2017177^T^).

## Data Availability Statement

The datasets presented in this study can be found in online repositories. The names of the repository/repositories andaccession number(s) can be found below: NCBI—MT036106, JABJXT000000000, JAEACP000000000.

## Author Contributions

CP and YL designed the experiments, provided the methods, and revised the manuscript. TM finished the manuscript and completed most of the experiments. HX helped to reconstructed and analyzed the gene trees. DB finished the fatty acid profiles. CL and MY collected the samples. All authors read and approved the final version of the manuscript.

## Conflict of Interest

The authors declare that the research was conducted in the absence of any commercial or financial relationships that could be construed as a potential conflict of interest.

## Publisher’s Note

All claims expressed in this article are solely those of the authors and do not necessarily represent those of their affiliated organizations, or those of the publisher, the editors and the reviewers. Any product that may be evaluated in this article, or claim that may be made by its manufacturer, is not guaranteed or endorsed by the publisher.

## Footnotes

1
https://lpsn.dsmz.de/family/rhodobacteraceae

2
https://lpsn.dsmz.de/genus/rhodobacter

3
https://www.ezbiocloud.net/identify

4
https://github.com/Ecogenomics/GtdbTk

5
http://ggdc.dsmz.de

6
https://github.com/dparks1134/CompareM

7
https://github.com/2015qyliang/POCP
