# Supplementary material for: Reclassification of 11 Members of the Family Rhodobacteraceae at Genus and Species Levels and Proposal of Pseudogemmobacter hezensis sp. nov
Source: Front Microbiol. 2022 Apr 13;13:849695. doi: 10.3389/fmicb.2022.849695 (PMC9044078; doi:10.3389/fmicb.2022.849695)
Supplement: Supplementary file 1 [file Data_Sheet_1.docx]

Supplementary Material

**Figure S1.** The relationship of AAI and POCP between *R. megalophilus* JA194^T^, *R. sediminicola* JA983^T^ and the related strains in the family *Rhodobacteraceae.* The green dot inside the dashed line represent the value of the two strains, the other dots inside the dashed line represent values between the two strains and strains in genus *Cereibacter*, and those outside represent the values between the two strains and strains in the related genera of the family *Rhodobacteraceae.* A total of 52 genomes were included in this analysis.

**Figure S2.** The relationship of AAI and POCP between *T. alkalilacus* DJC^T^, *T. sedimini*s DRYC-M-16^T^, *T. algicola* ETT8^T^ and the related strains in the family *Rhodobacteraceae.* The dots inside the dashed line represent the values between the three strains, and those outside represent the values between the three strains and strains in the related genera of the family *Rhodobacteraceae.* A total of 52 genomes were included in this analysis.

**Figure S3.** The relationship of AAI and POCP between *T. piscis* K13M18^T^, *T. aquatica* RCRI19^T^, *X. soli* ZQBW^T^, *R. flagellatus* SYSU G03088^T^, *R. thermarum* YIM 73036^T^and the related strains in the family *Rhodobacteraceae.* The dots inside the dashed line represent the values between the five strains, and those outside represent the values between the five strains and strains in the related genera of the family *Rhodobacteraceae.* The dots inside the solid line represent the three species of *Tabrizicola oligotrophica*,*Fuscovulum blasticum* and *Gemmobacter aestuarii*. A total of 52 genomes were included in this analysis.

**Figure S4.** Polar lipid profile of strain D13-10-4-6^T^ separated and detected by two-dimensional thin-layer chromatography and spraying with a molybdatophosphoric acid reagent, respectively. PME, phosphatidylmethylethanolamine; DPG,disphosphatidylglycerol; PE, phosphatidylethanolamine; PG, phosphatidylglycerol; PC, phosphatidylcholine; PL, phospholipid; AL, aminolipid; L, unidentifiedlipid.

 **Figure S5.** The relationship of AAI and POCP between *T. fusiformis* SY72^T^, *T. oligotrophica* KMS-5^T^ and the related strains in the family *Rhodobacteraceae.* The dots represent the values between the two strains, and the two strains with strains in the related genera of the family *Rhodobacteraceae.* A total of 52 genomes were included in this analysis.

**Table S1.** Genome features of the strains in this study.

| **Strain** | **Accession number** |
| --- | --- |
| strain D13-10-4-6^T^ | JABJXT000000000 |
| *Xinfangfangia humi* IMT-291^T^ | UXAW00000000 |
| *Pseudogemmobacter bohemicus* Cd-10^T^ | QNHG00000000 |
| *Xinfangfangia soli* ZQBW^T^ | JAEACP000000000 |
| *Cereibacter azotoformans* KA25^T^ | QAOT00000000 |
| *Cereibacter changlensis* DSM 18774^T^ | QKZS00000000 |
| *Cereibacter johrii* JA192^T^ | PZZW00000000 |
| *Cereibacter ovatus* JA234^T^ | OAOQ01000000 |
| *Cereibacter sphaeroides* 2.4.1^T^ | WSNV00000000 |
| *Cypionkella_psychrotolerans* PAMC 27389^T^ | LGIC00000000 |
| *Falsigemmobacter faecalis* YIM 102744-1^T^ | RRAZ00000000 |
| *Falsigemmobacter intermedius* 119/4^T^ | SBLC00000000 |
| *Falsirhodobacter deserti* W402^T^ | JQHS00000000 |
| *Fuscovulum blasticum* DSM 2131^T^ | PZKE00000000 |
| *Gemmobacter aestuarii* CC-PW-75^T^ | SSND00000000 |
| *Gemmobacter aquarius* HYN0069^T^ | CP028918 |
| *Gemmobacter aquaticus* A19^T^ | VOAK00000000 |
| *Gemmobacter_aquatilis* DSM 3857^T^ | FOCE00000000 |
| *Gemmobacter caeni* CGMCC 1.7745^T^ | VLLH00000000 |
| *Gemmobacter caeruleus* N8^T^ | VKKX00000000 |
| *Gemmobacter lutimaris* YJ-T1-11^T^ | QXXQ00000000 |
| *Gemmobacter* *serpentinus* HB-1^T^ | WCHR00000000 |
| *Gemmobacter* *tilapiae* KCTC 23310^T^ | BMYJ00000000 |
| *Phaeovulum veldkampii* DSM 11550^T^ | NHSP00000000 |
| *Phaeovulum vinaykumarii* JA123^T^ | OBMN00000000 |
| *Haematobacter massiliensis* CCUG 47968^T^ | JGYG00000000 |
| *Haematobacter missouriensis* CCUG 52307^T^ | JFGS00000000 |
| *Paenirhodobacter enshiensis* DW2-9^T^ | JFZB00000000 |
| *Pseudorhodobacter antarcticus* CGMCC 1.10836^T^ | FOCO00000000 |
| *Pseudorhodobacter aquimaris* KCTC 23043^T^ | LGHS00000000 |
| *Pseudorhodobacter ferrugineus* DSM 5888^T^ | ATVN00000000 |
| *Pseudorhodobacter wandonensis* KCTC 23672^T^ | LGHT00000000 |
| *Rhodobacter aestuarii* JA296^T^ | QAXT00000000 |
| *Rhodobacter capsulatus* DSM 1710^T^ | QKZO00000000 |
| *Rhodobacter* *flagellatus* SYSU G03088^T^ | VMDU00000000 |
| *Rhodobacter* *megalophilus* DSM 18937^T^ | FZOV00000000 |
| *Rhodobacter* *sediminicola* JA983^T^ | VDEK00000000 |
| *Rhodobacter* *tardus* CYK-10^T^ | JAABNR000000000 |
| *Rhodobacter* *thermarum* YIM 73036^T^ | QMJY00000000 |
| *Rhodobacter maris* JA276^T^ | OBMT00000000 |
| *Rhodobacter viridis* JA737^T^ | QJTK00000000 |
| *Sinirhodobacter* *ferrireducens* CCTCC AB2012026^T^ | SAVB00000000 |
| *Sinirhodobacter* *hankyongi* BO-81^T^ | RCHI00000000 |
| *Sinirhodobacter* *huangdaonensis* CGMCC 1.12963^T^ | SAVA00000000 |
| *Sinorhodobacter* *populi* SK2B-1^T^ | SAUZ00000000 |
| *Tabrizicola algicola* ETT8^T^ | JAAIKE010000000 |
| *Tabrizicola alkalilacus* DJC^T^ | QWEY00000000 |
| *Tabrizicola aquatica* RCRI19^T^ | PJON00000000 |
| *Tabrizicola fusiformis* SY72^T^ | JABUHN000000000 |
| *Tabrizicola oligotrophica* KMS-5^T^ | JAAIVJ000000000 |
| *Tabrizicola* *piscis* K13M18^T^ | CP034328 |
| *Tabrizicola_sediminis* DRYC-M-16^T^ | RPEM00000000 |
| *Aquidulcibacter paucihalophilus* TH1-2^T^ | NCSQ00000000 |

**Table S2.** The values of AAI (%, lower diagonal) and POCP (%, upper diagonal) among strain D13-10-4-6^T^, *P. bohemicus* Cd-10^T^, *X. humi* IMT-291^T^ and the strains in the related genera of the family *Rhodobacteraceae*.

Strain: 1, D13-10-4-6^T^; 2, *Pseudogemmobacter bohemicus* Cd-10^T^; 3, *Xinfangfangiahumi* CIP 111625^T^; 4, *Xinfangfangiasoli* ZQBW^T^; 5, *Cereibacterazotoformans* KA25^T^; 6, *Cereibacterchanglensis* DSM 18774^T^; 7, *Cereibacterjohrii* JA192^T^; 8, *Cereibacterovatus* JA234^T^; 9, *Cereibactersphaeroides* 2.4.1^T^; 10, *Cypionkellapsychrotolerans* PAMC 27389^T^; 11, *Falsigemmobacterfaecalis* YIM 102744-1^T^; 12, *Falsigemmobacterintermedius* 119/4^T^; 13, *Falsirhodobacterdeserti* W402^T^; 14, *Fuscovulumblasticum* DSM 2131^T^; 15, *Gemmobacteraestuarii* CC-PW-75^T^; 16, *Gemmobacteraquarius* HYN0069^T^; 17, *Gemmobacteraquaticus* A1-9^T^; 18, *Gemmobacteraquatilis* DSM 3857^T^; 19, *Gemmobactercaeni* DSM 21823^T^; 20, *Gemmobactercaeruleus* N8^T^; 21, *Gemmobacterlutimaris* YJ-T1-11^T^; 22, *Gemmobacterserpentinus* HB-1^T^; 23, *Gemmobactertilapiae* KCTC 23310^T^; 24, *Haematobactermassiliensis* CCUG 47968^T^; 25, *Haematobactermissouriensis* CCUG 52307^T^; 26, *Paenirhodobacterenshiensis* DW2-9^T^; 27, *Phaeovulumveldkampii* DSM 11550^T^; 28, *Phaeovulumvinaykumarii* DSM 18714^T^; 29, *Pseudorhodobacterantarcticus* CGMCC 1.10836^T^; 30, *Pseudorhodobacteraquimaris* KCTC 23043^T^; 31, *Pseudorhodobacterferrugineus* DSM 5888^T^; 32, *Pseudorhodobacterwandonensis* KCTC 23672^T^; 33, *Rhodobacteraestuarii* JA296^T^; 34, *Rhodobactercapsulatus* DSM 1710^T^; 35, *Rhodobacterflagellatus* SYSU G03088^T^; 36, *Rhodobactermaris* JA276^T^; 37, *Rhodobactermegalophilus* DSM 18937^T^; 38, *Rhodobactersediminicola* JA983^T^; 39, *Rhodobactertardus* CYK-10^T^; 40, *Rhodobacterthermarum* YIM 73036^T^; 41, *Rhodobacterviridis* JA737^T^; 42, *Sinirhodobacterferrireducens* CCTCC AB2012026^T^; 43, *Sinirhodobacterhankyongi* BO-81^T^; 44, *Sinirhodobacterhuangdaonensis* CGMCC 1.12963^T^; 45, *Sinorhodobacterpopuli* SK2B-1^T^; 46, *Tabrizicolaalkalilacus* DJC^T^; 47, *Tabrizicolaaquatica* RCRI19^T^; 48, *Tabrizicolapiscis* K13M18^T^; 49, *Tabrizicolasediminis* DRYC-M-16^T^; 50,*Tabrizicola fusiformis* SY72^T^; 51,*Tabrizicola oligotrophica* KMS-5^T^; 52,*Tabrizicola algicola* ETT8^T^.
